# How Safe to Eat Are Raw Bivalves? Host Pathogenic and Public Health Concern Microbes within Mussels, Oysters, and Clams in Greek Markets

**DOI:** 10.3390/foods10112793

**Published:** 2021-11-13

**Authors:** Athanasios Lattos, Ilias Chaligiannis, Dimitrios Papadopoulos, Ioannis A. Giantsis, Evanthia I. Petridou, George Vafeas, Alexandra Staikou, Basile Michaelidis

**Affiliations:** 1 Laboratory of Animal Physiology, Department of Zoology, Faculty of Science, School of Biology, Aristotle University of Thessaloniki, 54124 Thessaloniki, Greece; ichaligiannis@bio.auth.gr (I.C.); dkpapado@bio.auth.gr (D.P.); michaeli@bio.auth.gr (B.M.); 2 Environmental Control and Research Laboratory, Region of Central Macedonia, 54625 Thessaloniki, Greece; astaikou@bio.auth.gr; 3 Hellenic Agricultural Organization-DEMETER, Veterinary Research Institute of Thessaloniki, Campus of Thermi, 57001 Thermi, Greece; vafeas@vri.gr; 4 Department of Animal Science, Faculty of Agricultural Sciences, University of Western Macedonia, 53100 Florina, Greece; 5 Laboratory of Microbiology and Infectious Diseases, Faculty of Veterinary Medicine, School of Health Science, Aristotle University of Thessaloniki, 54124 Thessaloniki, Greece; epetri@vet.auth.gr; 6 Department of Zoology, School of Biology, Faculty of Science, Aristotle University of Thessaloniki, 54124 Thessaloniki, Greece

**Keywords:** foodborne pathogens, enteropathogenic diseases, vibrionaceae, *Photobacterium damselae*, food safety, public health

## Abstract

Raw-bivalves consumption is a wide trend in Mediterranean countries. Despite the unambiguous nutritional value of seafood, raw consumption of bivalves may involve risks that could pose a significant threat to consumers’ health. Their filter-feeding behavior is responsible for the potential hosting of a wide variety of microorganisms, either pathogenic for the bivalves or public health threats. Under this prism, the current study was conducted in an effort to evaluate the risk of eating raw bivalves originating from the two biggest seafood markets in Thessaloniki, the largest production area of bivalves in Greece. Both microbiological and molecular methodologies were applied in order to assess the presence of various harmful microbes, including noroviruses, *Bonamia*, *Marteilia*, *Esherichia coli*, *Salmonella,* and *Vibrio*. Results indicated the presence of several *Vibrio* strains in the analyzed samples, of which the halophilic *Vibrio harveyi* was verified by 16S rRNA sequencing; other than this, no enteropathogenic Vibrio spp. was detected. Furthermore, although *Esherichia coli* was detected in several samples, it was mostly below the European Union (EU) legislation thresholds. Interestingly, the non-target *Photobacterium damselae* was also detected, which is associated with both wound infections in human and aquatic animals. Regarding host pathogenic microorganisms, apart from *Vibrio harveyi*, the protozoan parasite *Marteilia refrigens* was identified in oysters, highlighting the continuous infection of this bivalve in Greece. In conclusion, bivalves can be generally characterized as a safe-to-eat raw food, hosting more bivalve pathogenic microbes than those of public health concern.

## 1. Introduction

Marine seafood has been an integral part of the human diet throughout humankind history and has been correlated since then with a healthy diet and the well-being of humans [1]. Fish and seafood play a key role in human nutrition due to their high content in essential nutrients [2]. Marine mollusk-bivalves are considered to be also a nutritious diet, as they contain high quality of protein and minerals, low lipid content, and high concentration of polyunsaturated fatty acids (PUFAs) [3]. PUFAs are essential for human health, cannot be synthesized by the human organism, and can be only supplied by external sources [4]. Specifically, consumption of eicosapentaenoic (EPA) acid has been associated with beneficial effects in cardiovascular system, while consumption of docosahexaenoic (DHA) acid has reported to play a key role in brain functions, photoreception function, and the reproductive system [5,6,7].

Marine bivalve-mollusk aquaculture in Greece is limited solely to Mediterranean mussel *Mytilus gallorpovincialis* farming, while the remaining commercial marine bivalve production (*Callista chione*, *Ostrea edulis*, and *Venus verrucosa*) intended for human consumption is provided to the supply chain as a result of fisheries [8]. Despite the great importance in their production and fisheries, marine bivalve harvesting suffers limitations and heavy losses alongside finfish aquaculture due to climate change [9]. Global climate change and its impacts have immediate effects on bivalve physiological functions and immune responses, making them vulnerable to opportunistic pathogens [10,11,12,13]. For instance, recently in Greece, *Vibrio* spp. decimated *Pinna nobilis* populations, with a synergistic effect on other microorganisms as well [14,15,16,17]. In this context, protozoan or protistan parasites, such as *Marteilia refringens* and *Bonamia ostreae,* respectively, have been detected during summer temperatures, causing heavy mortalities in flat oyster *O. edulis* populations, limitations due to lowering physiological functions, and also mortalities in cultured mussels, *M. galloprovincialis,* in Thermaikos gulf, Thessaloniki [18,19,20].

Although raw seafood consumption is more widely assigned to preparations such as sushi, incorporating fish like tuna, blue marlin, and swordfish, consumption of raw bivalves is a common trend as well. Nevertheless, marine bivalves can act as vectors of foodborne diseases involving safety risks, reinforced by the tendency of the consumers to consume them raw, steamed, or generally slightly processed [21,22,23]. Under this prism and despite the occurrence of bivalve-opportunistic microorganisms limiting bivalve production and farming, foodborne emerging diseases constitute a matter of crucial public health importance [24]. Marine bivalves may act as vectors of foodborne pathogens, such as bacteria (*Salmonella*, *Campylobacter*, pathogenic forms of *E. coli*, *Vibrio* spp.), viruses (norovirus, hepatitis A virus), and parasites (*Giardia*, *Cryptosporidium*) [25], whereas safety risks are amplified by the tendency of the consumers to consume them raw or slightly processed.

The bacterial human pathogens could be classified as allochthonous of fecal origin (pathogenic *E. coli*, *Salmonella*) or allochthonous from aquatic environment (*Aeromonas, Pseudomonas*) and as indigenous, such as *Vibrio* [26]. In the European Union, the enumeration of *E. coli* as an indicator of fecal contamination is the standard way to estimate the associated potential risk to human health from all waterborne enteric pathogens [27]. Moreover, *E. coli* includes strains that can be pathogenic to humans [28] and cause gastroenteritis in humans after consumption of contaminated seafood [29].

Non-indigenous pathogen bacteria of the genus *Salmonella* spp. are introduced into the aquatic environment via inappropriate disposal of human wastes, agricultural runoffs, or sewage discharges [30], while indigenous bacteria are naturally occurring organisms in the marine environment, mainly belonging to the family *Vibrionaceae* [25]. Although salmonellosis is considered the second most commonly reported gastrointestinal infection and an important cause of foodborne outbreaks in the EU [31], and despite the presence in the aquatic environment [32], the risk of foodborne diseases associated with shellfish consumption is very low [33].

On the other hand, *Vibrio* spp., such as *Vibrio parahaemolyticus*, *Vibrio cholerae*, *Vibrio vulnificus,* and *Vibrio alginolyticus,* are considered to be extremely dangerous for public health [34]. *V. parahaemolyticus* is widely distributed in marine and estuarine environments and can cause foodborne disease with consumption of raw or slightly cooked marine bivalves [35,36]. However, all strains of *V. parahaemolyticu,* except those that bear the two pathogenic genes *tdh* and *trh* do not pose a threat to public health [37]. Among all known *V. cholerae* serotypes, only two (O1 and O139) possess the virulence genes required to cause diseases in public health [38,39]. Transmission of *V. cholerae* from environmental reservoirs to humans is associated with contaminated seawater or raw seafood consumption [39]. Genes associated with disease pathogenesis in humans are *ctxA*, *ompU,* and *toxR* [39]. *V. vulnificus* inhabits brackish aquatic environments and especially tropical and subtropical environments [40]. *V. vulnificus* causes fatal wound infections, sepsis, and food-related infections when comes in contact with skin lesions or is swallowed though consumption of raw seafood [39,40]. This bacterium exhibits an invasive character and has been detected to cause health problems worldwide [41,42]. Halophilic *V. alginolyticus* is considered to be an inhabitant of both marine and estuarine aquatic environments [43,44]. *V. alginolyticus* is hosted in mussels, but it has been also detected in finfish and in other seafood [45]. This halophilic bacterium has been known to cause diseases in aquatic animals; however, it can also carry genes that make it a threat to human health [46,47,48,49].

Furthermore, food-borne viruses represent an important and emerging problem for food safety and public health. According to a report by European Food Safety Authority (EFSA) (2015), in 2014, viruses were the most commonly detected (20.4%) causative agent in food-borne outbreaks [50]. Norovirus (NoV), family Caliciviridae, is considered the leading cause of acute gastroenteritis in children and adults in many developed countries [51,52,53], causing sudden diarrhea and vomiting in millions of cases worldwide annually [54] and more than 200,000 deaths worldwide each year, especially in children [55]. NoVs are non-enveloped viruses with a 7.5–7.7-kb positive-sense single-stranded RNA genome containing three open-reading frames [56]. Norovirus classification scheme was updated, and NoVs are classified into ten genogroups (GI-GX) and forty-eight genotypes [57,58]. NoV genogroups that infect humans are I (NoV GI), II (NoV GII), and rarely IV (NoV GIV). NoV GII.4 variants (such as GII.4 Sydney, GII.4 New Orleans, GII.4 Hong Kong) are responsible for 80% of the disease outbreaks [59]. According to World Health Organization (WHO), <10 virions are enough to cause infection and gastroenteritis in adults. Filter-feeding shellfish are an important vehicle for transmission of norovirus (NoV) when grown in sewage-polluted water [60] since they are able of accumulating and concentrating pathogens present in the water [61]. Thus, consumption of raw shellfish is a major risk factor for food-borne outbreaks [61,62,63,64]. NoV illnesses due to shellfish consumption present a seasonal pattern, with a peak incidence usually during the wintertime [65,66].

Taken all together, the main objective of this research is to investigate the prevelance of foodborne pathogens and host-associated parasites in bivalves consumed as raw food. To achieve these goals, the presence of a wide range of public health risks and bivalve pathogenic microorganism taxa, including bacteria, protozoan parasites, and viruses, were systematically monitored from two seafood markets in Greece on a monthly basis.

## 2. Materials and Methods

### 2.1. Sampling

Samplings for the needs of the current research were performed on a monthly basis from the two biggest seafood markets in the Regional Unit of Thessaloniki (Region of Central Macedonia, Greece). A total number of 87 samples were obtained from both seafood markets. The first sampling site (sampling site 1) was in the city center of Thessaloniki, namely the Kapani Market, and the second seafood market (sampling site 2) was in Nea Michaniona, a small town inside the Regional Unit of Thessaloniki (Nea Michaniona Market). Each sampling of a specific marine bivalve consisted of approximately 25 individuals (Table 1). Of the 25 individuals in each species, 100 g of the digestive gland of the sampling were stored in the freezer (−23 °C) for microbiological analysis, 50 g of digestive glands were stored in deep freezing temperatures (−80 °C) to investigate the presence of foodborne viruses, and the rest of the tissues alongside the intervalvular fluid were kept for further microbiological processes.

### 2.2. E. coli and Salmonella spp. Detection

All the bivalve mollusks were analyzed for the detection of *Salmonella* spp. according to the International Organization for Standardization (ISO) 6579-1:2017 [67]. Twenty-five grams of flesh and intervalvular fluid were weighted, and 225 mL of Buffered Peptone Water (BPW) were added and homogenized. After incubation at 37 °C for 18 h, 0.1 mL of the pre-enriched culture were plated in 3 equally spaced spots onto the surface of Modified Semi-solid Rappaport Vassiliadis agar (MSRV) (OXOID, Basingstoke, UK) and incubated not inverted at 41.5 °C for 24 h, while 1 mL of the same pre-enrichment culture was added to 10 mL of the Muller–Kauffman tetrathionate/novobiocin broth (MKTTn-Biolife, Italian S.r.L, Milano, Italy) and incubated at 37 °C for 24 h. Negative MSRV plates were re-incubated for 24 h and examined for the presence of white grey colonies with a turbid zone around the spot. Suspected colonies from the MSRV plates and a loop from MKTTn were spread onto the surface of Xylose Lysine Deoxycholate agar (XLD) (Merck, Darmstadt, Germany) and RAMBACH (Merck, Darmstadt, Germany) agar plates, incubated at 37 °C for 24 h, and the presence of the growth of black-center-with-reddish-transparent-zone colonies in XLD agar plates and the presence of pink-red-colored colonies in RAMBACH agar plates was examined. Enumeration of *E. coli* in bivalve mollusk samples was performed using the multiple tube method with the 5-tubes-3-dilutions test according to the ISO/TS 16649-3: 2015 [68]. One hundred (100) grams of flesh and intervalvular fluid was added to 200 mL of Peptone water, and the mix was homogenized using a stomacher homogenizer for 2 min. Then, 30 mL of the mix were added to 70 mL of Peptone Water, resulting in a 1:10 dilution. Next, 10 mL of this homogenate were added in 90 mL of Peptone Water, resulting in a 1:100 dilution. Afterwards, 10 mL from the 10^−1^ homogenate was inoculated into five tubes of double strength of Minerals Modified Glutamate Broth (MMGB). Five tubes of single-strength MMGB were inoculated with 1 mL of the 10^−1^ homogenate, while the other five single-strength tubes were inoculated with 1 mL of a 10^−2^ homogenate per tube. All the tubes were incubated at 37 °C for 24 h. *E. coli* confirmation was performed by culturing the positive tubes (tubes showing acid production) on the Tryptone Bile X-Glucuronide agar (TBX) plates and by incubating them at 44 °C for 24 h. The presence of β-glucuronidase-positive *E. coli* was indicated by the growth of blue or blue-green colonies. The number of *E. coli*/100 g was determined using the MPN tables [69]. The lowest detectable concentration of *E. coli* was 20 cfu/100 g.

### 2.3. Microbiological Culture of Tissues and Molecular Identification of Cultures

Detection of potentially enteropathogenic *Vibrio* spp. was performed according to the ISO 21872-1:2007 [70] and ISO 21872-2:2007 [71] for the detection of *Vibrio parahaemolyticus* and *Vibrio cholerae* and for the detection of species other than *Vibrio parahaemolyticus* and *Vibrio cholerae*, respectively. Specifically, for the detection of *Vibrio parahaemolyticus* and *Vibrio cholerae*, 25 g of flesh and intervalvular water of each sample that was kept deep-frozen was aseptically placed and weighted in a sterile stomacher bag, and 225 mL of Alkaline Saline Peptone Water (ASPW) adjusted at pH 8.6 were added. After homogenization, the samples were incubated aerobically at 37 °C for 6 h. From the enrichment culture, a loop of 10 μL was transferred and spread onto both selective Thiosulfate-Citrate Bile salts-Sucrose (TCBS) agar (Biokar Diagnostics, Allonne, France) and ChromID Vibrio agar (bioMerieux, Craponne, France) and incubated at 37 °C for 24 h. All plates were then examined for the presence of smooth blue-green or yellow colonies on TCBS agar and smooth pink or blue colonies on ChromID. Respectively, for the detection of species other than *Vibrio parahaemolyticus* and *Vibrio cholerae,* 1 mL from the above first enriched culture was transferred to a second enrichment of 10 mL of ASPW, and 18 h of incubation at 37 °C followed. Then, a loop from the inoculum was inoculated onto the surface of a TCBS agar plate to allow the growth of well-isolated colonies. To obtain pure colonies for further molecular identification, 5 suspected, e.g., *Vibrio* spp., colonies (smooth, green or yellow) were inoculated for 24 h at 37 °C onto the surface of Saline Nutrient Agar made with 5 g/L meat extract, 3 g/L peptone, 10 g/L NaCl, and 12 g/L agar adjusted at pH 7.2.

For molecular identification of the cultured microorganisms, DNA extraction of bacterial pure cultures, Polymerase Chain Reaction (PCR) amplification of a conserved partial 16S rRNA gene, and agarose gel electrophoresis for visualization of PCR products were carried out exactly as described in our previous study [16].

### 2.4. Molecular Examination for the Presence of Marteilia, Bonamia, and Vibrio spp.

Homogenization of each specimen was performed manually with the use of piston pellets within 1.7 mL microcentrifuge tubes. Approximately 20 mg of homogenized digestive glands of each species were subjected for DNA isolation. The DNeasy Blood and Tissue kit (Qiagen, Hilden, Germany) was utilized for isolation of genomic DNA, following the instructions of the manufacturer company. The detection of the protozoan parasites *Martelia* spp. was assayed in all oysters and mussels applying a conventional PCR with the primer pair SS2/SAS2 as described by LeRoux [72]. The potential presence of the *Bonamia* sp. protistan parasite was examined in oysters applying a PCR with primers BON-1310F and BON-745R [73]. For the detection and identification of *Vibrio parahaemolyticus*, *Vibrio cholerae*, *Vibrio vulnificus,* and *Vibrio alginolyticus*, the multiplex PCR developed by Xu et al. [74] was applied in all specimens, with a *Vibrio alginolyticus* strain identified in a previous study from our lab included as a positive control [20]. All PCR reactions were performed using the FastGene Taq 2x Ready Mix (NIPPON Genetics, Tokyo, Japan), with conditions as described in the aforementioned studies, in 20 μL volumes.

### 2.5. Sequencing and Phylogenetic Analysis

Successfully amplified products of all performed PCRs, i.e., positive samples, were purified using the Nucleospin gel and pcr clean-up kit (Macherey-Nagel, Düren, Germany) following the manufacturer’s recommended protocol and sequenced in both directions using the corresponding forward and reverse primer. Sequences were read, edited, and aligned in the software MEGA [75] and phylogenetically analyzed in comparison to closely related ones retrieved from GenBank after search in the Basic Local Alignment Search Tool (BLAST) in National Center for Biotechnology Information (NCBI) website. Maximum likelihood dendrograms were constructed in the same software applying 1000 bootstrap iterations.

### 2.6. Molecular Investigation of Foodborne Viruses

Total RNA was extracted from the homogenised digestive gland of each collected specimen using NucleoZOL Reagent (Macherey-Nagel, Düren, Germany), according to the the manufacturer’s instructions. Briefly, digestive glands (50 mg per sample) were homogenized by pestling in 1 mL NucleoZOL. RNAase-free water was added to the lysate, and samples were certifuged. Afterwards, isopropanol was added to the supernatant for RNA precipitation and was followed by centrifugation and two ethanol washes. RNA pellet was dissolved in 60 μL nuclease-free water.

The presence of NoV GI and NoV GII were investigated through the TaqMan reverse transcription real-time PCR surveys developed by Kageyama et al. [76]. Approximately 100 ng of extracted RNA were used as template in 10-μL total volume reactions that were performed in an Eco 48 real-time PCR system (Cole-Parmer Antylia Scientific, Vernon Hills, USA), using the One Step PrimeScript III RT-qPCR Mix (TAKARA, Kusatsu Japan), containing 5 μL 2X RT-qPCR Mix, 0.3 pmol of each forward and reverse primer (COG1F-COG1R for NoV GI and COG2F-COG2R for NoV GII [76]), 0.2 μL probe (RING1a-TP for NoV GI and RING2-TP for NoV GII [76]), and ultrapure water up to the final volume. Reactions were performed following a regime that consisted of 95 °C for 3 min and 40 cycles of 95 °C for 15 s and 60 °C for 30 s, whereas genomic NoV GI and NoV GII RNA extracted from domestic animal fecal samples was used as positive control.

## 3. Results

Bacteriological data showed that all samples were negative for *Salmonella* spp., whereas 22 of them (25.3%) were contaminated with *E. coli*. In 63.6% of the samples that were positive for *E. coli,* the bacterial counts were 20 cfu/100 g. Only one sample showed *E. coli* values higher than 230 cfu/100 g (Table 2), which is the value allowed by current legislation. The number of *E. coli* detected in the Kapani fish market ranged from 20 cfu/100 g to 2400 cfu/100 g, while that in the Nea Michaniona fish market ranged from 20 cfu/100 g to 92 cfu/100 g.

**Figure 1:**
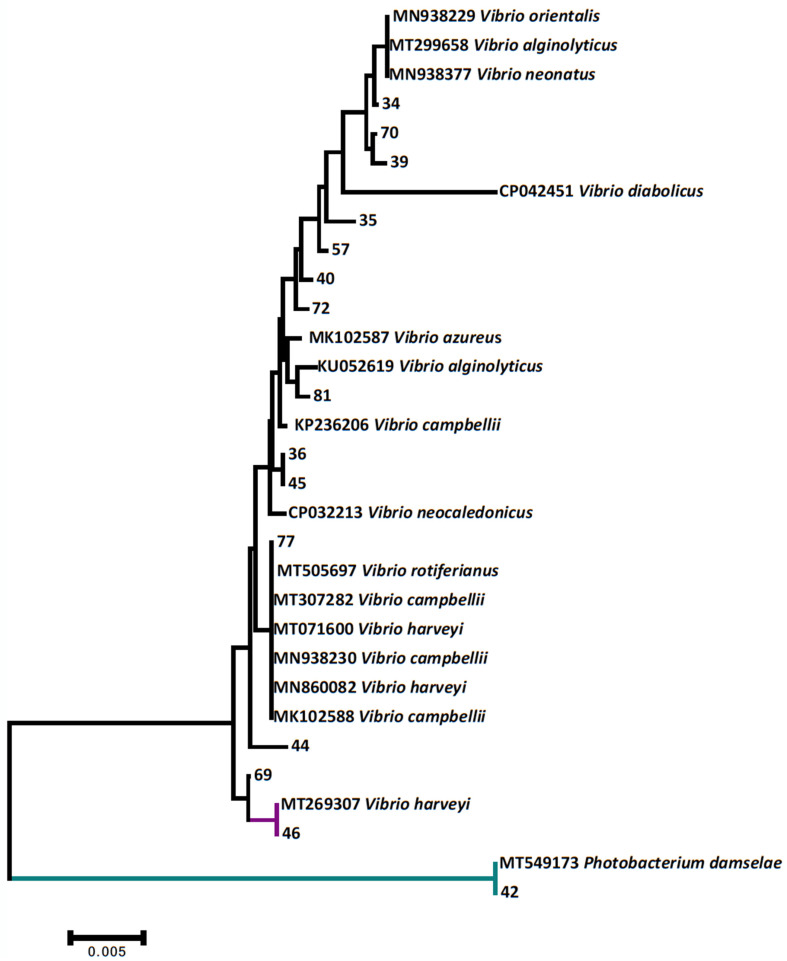
Maximum likelihood phylogenetic dendrogram of the detected cultured bacteria in comparison to closely related haplotypes retrieved from GenBank, based on the 16 s rRNA gene. With the exception of *Vibrio neocaledonicus*, all the *Vibrio* taxa included in the dendrogram are considered validly published under the International Code of Nomenclature of Bacteria.

**Figure 2:**
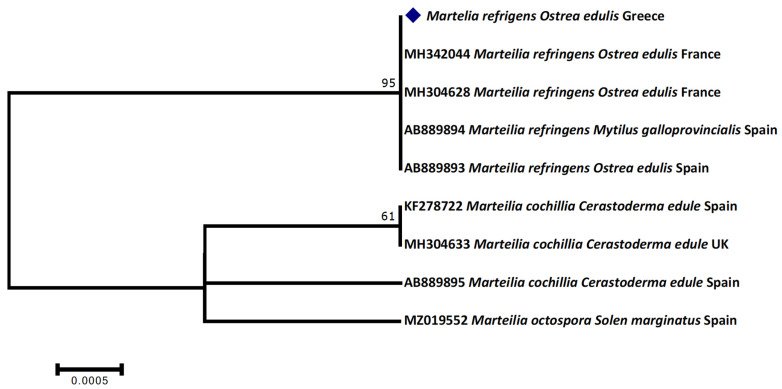
Maximum likelihood phylogenetic tree of the *Marteilia refrigens* strain detected hosting *Ostrea edulis* in comparison to congeneric strains from GenBank.

While no *Vibrio parahaemolyticus*, *Vibrio cholerae*, *Vibrio vulnificus,* and *Vibrio alginolyticus* was detected by the multiplex PCR applied, microbiological cultures indicated that 44 of the analyzed specimens were positive to *Vibrio* spp., confirmed by sequencing of the 16S rRNA. However, with the exception of two samples clearly identified as *V. harveyi*, the majority of those strains were clustered among a wide range of Vibrios, including *V. campbellii*, *V. orientalis*, *V. azureus,* and *V. neocaledonicus*, the latter of which is not validly published in the List of Prokaryotic Names with Standing in Nomenclature (https://lpsn.dsmz.de/, accessed on 24 October 2021) and hence could not be securely identified (Figure 1). Interestingly, based on the phylogenetic analysis, one *Ostrea edulis* examined specimen was positive for *Photobacterium damselae*. Regarding the pathogenic parasites, although no specimen was positive for *Bonamia* sp., *Marteilia* sp. was detected as hosting *O. edulis*, identified as *Marteilia refrigens* according to sequencing and phylogenetic analysis (Figure 2).

Finally, all the samples obtained from the seafood markets were also investigated for the presence of the foodborne enteric pathogenic viruses NoV GI and NoV GII. However, NoV was not found in any of the samples analyzed.

## 4. Discussion

The aim of this study was to determine the prevalence of potentially human pathogens, such as *Vibrio* spp., *Salmonella* spp., *E. coli,* and noroviruses, in mollusk-bivalves collected from the two sea food markets in the area of Thessaloniki. Among the detected microbes in the examined bivalves, Vibrio bacteria exhibited the highest prevalence. *Vibrio* is a genus of gram-negative, rod-shaped bacteria that can be found in a wide range of marine and estuarine environments, whereas at least 12 species belonging in this genus are considered to cause human infections [77]. Three major pathogens that pose a public health threat, *V. vulnificus*, *V. parahaemolyticus,* and *V. cholerae,* are affected by the aforementioned factors, causing millions of infections and thousands of deaths per year worldwide [78]. Regarding the remaining pathogenic species within this genus, infections are mostly caused by halophilic species that thrive in saltwater environments [77]. These infections are occasionally associated with exposure of open wounds to these pathogens o more often, with consumption of unprocessed or raw seafood [79]. In our study, although a large number of the detected Vibrios could not be reliably identified at species level (Figure 1), the presence of the three aforementioned ones can be clearly excluded by both molecular techniques applied, namely the sequencing of the 16S rRNA gene and the multiplex PCR [75].

On the other hand, 16s rRNA phylogenetic analysis demonstrated the identification of *V. harveyi* (Figure 1). This bacterium is considered to be a harmful microorganism for both aquatic animals and public health [77,80]. *V. harveyi* has been associated with several systemic aquatic animal diseases [81]. This gram-negative bacterium has been characterized as the causative agent of mortalities in larval stages of shrimp species *P. monodon* and *P. vannamei,* resulting in heavy losses in shrimp aquaculture [82,83,84]. It is also considered as the etiological agent of mortalities of ark clams, *Scapharca broughtonii*, and the disease agent in the European abalone, *Haliotis tuberculate,* and the Pacific oyster, *C. gigas* [80,85,86]. Additionally, it has been isolated from diseased fish originated from aquacultures in China as well as from both major species *D. labrax* and *S. aurata,* farmed in Mediterranean Sea, whereas in rearing facilities, it poses a threat for farmed aquatic animals [87,88,89]. Apart from the adverse impacts in aquatic animals, *V. harveyi* has been recently associated with sporadic cases of human infections as well [77]. The infection cases with this thermo-dependent bacterium are associated with transmission of the pathogen through open wounds [90].

Interestingly, apart from *Vibrio* spp., *Photobacterium damselae subs. damselae* was also identified, which is a marine autochthonous bacterium belonging to Vibrionaceae family, infecting both marine animals and humans [91]. Strains of this pathogen have been isolated from water samples originating from marine and estuarine environments, from marine animals and symbiotic microorganisms, as well as from seafood [91,92,93]. Despite its pathogenic nature for marine animals, it is considered to belong in the symbiotic microbiome of carcharhinid sharks [94]. *P. damselae* was firstly isolated in 1971 as a causative agent of a human infectious disease and was assigned its taxonomic name, *Vibrio damsela,* due to its isolation from skin ulcers in the marine fish damselfish (*Chromis punctipinnis*) [91,95]. Afterwards, *Vibrio damselae* was proposed to be taxonomically reconstructed as *Photobacterium damsel,* owing to phenotypic and genetic studies that indicated its similarity to the species belonging to the genus *Photobacterium* [96]. The close genetic relation of *Photobacterium damselae* with *Pasteurella piscicida*, i.e., the etiological agent of Pasteurellosis in fish, based on molecular analyses, resulted to the identical taxonomy of these two bacteria with only differentiated subspecies status, namely *Photobacterium damselae* subsp. *damselae* and *Photobacterium damselae* subsp. *piscicida,* respectively [97].

Despite its importance in disease pathogenesis of wild aquatic animals, *Photobacterium damselae* subsp. *damselae* constitutes an important factor for disease pathogenesis in farmed aquatic animals as well [98]. Alongside the *Photobacterium damselae* subsp. *piscicida*, these two *Photobacterium* species play a crucial role in mortalities of farmed aquatic species in mariculture worldwide [98,99]. *P. damselae* subsp. *piscicida* can infect a wide variety of cultured marine species worldwide, including the Japanese amberjack (*Seriola quinqueradiata*), the European gilthead sea bream (*Sparus aurata*), sea bass (*Dicentrarchus labrax*) and the flatfish (*Solea senegalensis* and *Solea solea*), the members of the family Moronidae farmed in the U.S.A (*Morone saxalitis*, *Morone americana*), the cobia (*Rachycentron canadum*), and the golden pompano (*Trachinotus ovatus*) from Taiwan and China, respectively [100,101,102]. On the other hand, *Photobacterium damselae* subsp. *damselae* has a more opportunistic behavior as a pathogen in warm- and cold-water aquatic animals, and stress factors, such as thermal stress, seem to be associated with disease development [103,104]. Recently, in a yearly epidemiological report, *Photobacterium damselae* subsp. was reported to cause skin ulcerations in farmed black rockfish (*Sebastes schlegeli*) in a mariculture in North China [105].

Regarding public health, *Photobacterium damselae* subsp. *damselae* is considered to be a serious threat, while no reports of human infections with *Photobacterium damselae* subsp. *piscicida* have been referred to our knowledge. Infections caused by *Photobacterium damselae* subsp. *damselae* are mostly results of wound exposure to saltwater or brackish water with the presence of the pathogen, while fewer reports associated with infection through ingestion of seafood and though urinary tract have been recorded [95,106,107,108,109,110]. *Photobacterium damselae* subsp. *damselae* can cause severe infections and fasciitis, which may result in fatal incidences for the patients despite prompt antibiotic treatment, and in addition, surgical removal of the infection is recommended in early stages [106,109,111,112].

Another important group of organisms dangerous for the public health and responsible for causing gastrointestinal infections in humans are the enteric viruses NoVGI and NoVGII. Bivalve mollusks are an important source of NoV contamination and have been linked to several outbreaks in humans in many countries [113,114,115]. In Greece, in line with our results, there is a lack of information regarding the NoV prevalence in harvested and commercialized shellfish. In 2012, the prevalence of different enteric viruses in commercial mussels was evaluated at the retail level in three European countries (Finland, Greece, and Spain). According to Diez-Valcarce et al. [116], no positive samples for NoV were detected in *M. galloprovincialis* in Greece although most of them were imported from third-party countries. These results are consistent with the findings of the present survey. The absence of the enteric noroviruses in bivalves collected from the two fish markets could indicate that they are marketed with an adequate depuration process in order to remove these pathogens or alternatively could be attributed to the difficulty of detecting these RNA viruses and the fact that the effectiveness of the RT–PCR method depends on two factors: the effectiveness of nucleic acid extraction and its purity [117].

Depuration of marine bivalves is a mandatory technique for the public health safety based on the expulsion of accumulated microorganisms into the surrounding water when they are placed in a natural environment with clean water [118]. Although it has been regulated by the European Union under certain terms in order to cleanse marine bivalves from public health-related microorganisms [119,120], the depuration process is not certified to clean up the target microorganism entirely [118]. Specifically, marine bivalve depuration may be not completely effective in cases with a high level of microbial load and in cases with public health viruses, such as HAV (hepatitis A virus) [121]. Practically, due to demand in the local markets, and on account of high exportation rates in foreign markets, many depuration plans adopt short-time depuration protocols in order to clear marine bivalves rapidly and afford them to food markets without taking into account the differential infection rates of shellfish species [120]. Optimization of depuration process should be adopted by the means of improved hygiene practices inside the plants, extensive clearance of bivalve epibionts before the depuration process, and selection of qualified staff alongside microbial surveillance from local authorities to reassure the consumers and boost the demand in local markets. Finally, public health awareness campaigns from local authorities in cooperation with scientists will reduce disease cases that prevent consumers from raw-eating habits.

In similar studies conducted in neighboring countries, the prevalence of enteric noroviruses was established as follows: 14–15% for NoV [122,123,124] and 34.4% for NoV [125] in Italy, 16% in Slovenia [126], and 10.5% in Albania [127]. Low prevalence of NoV or even absence of contamination in bivalve mollusks has also been reported in countries such as Australia, India, Japan, the Netherlands, and Norway [115,128]. Higher prevalence was reported at the Spanish coast of the Mediterranean Sea [129], and the highest prevalence (76.2%) has been recorded in UK-based harvesting areas [130].

According to the European Union, the risk of fecal contamination from human and animal sources in bivalve mollusks is determined by the concentration of *E. coli* in samples taken from production areas [131]. In our study, *E. coli* was detected in the Nea Michaniona fish market with values ranging from 20 cfu/100 g to 92 cfu/100 g and in the Kapani fish market with values from 20 cfu/100 g to 2400 cfu/100 g. Only one sample showed an *E. coli* value higher than 230 cfu/100 g, which was the highest level of *E. coli* that was detected. The occurrence of such a high load of *E. coli* in *M. galloprovincialis* sold at the fish market could be explained due to the lack of good hygiene practices, including, among other things, premises cleaning and sanitizing and personal hygiene of the handler, or due to contamination during distribution of the bivalve mollusks [132].

Finally, the detection of *M. refringens* hosting *O. edulis* can be characterized as an unsurprising finding, as it continues to parasitize mussels and oysters in Greece. *M. refringens* is a protozoan parasite of the phylum cercozoan and order Paramyxida causing infection of the digestive gland of the marine bivalves and downregulation of the physiological processes, resulting lower growth rates and even losses in farms and in natural beds [20,133,134]. Detection rates of this parasite are higher in summer months, where temperatures demonstrate higher values in comparison with the winter months, and this phenomenon is amplified due to global climate warming [135,136,137]. Nevertheless, the Aber disease is not yet fully understood [136]. Despite its high mortality ratio, and especially in summer months, no cases have been reported as causing infections to public health.

In conclusion, based on the present study, raw bivalves can be generally characterized as quite safe to eat. In particular, with very few exceptions, the major microbial load within marine bivalves in Greece consists of host pathogenic bacteria, protozoans or other parasites, and a far lesser extent of public health harmful microorganisms. Hence, despite the microbial load detected that is in line with a recently published review paper [137], our data suggest that Greek bivalves are microbiologically safe for raw consumption, and the sector of marine bivalve farming and fisheries, when proper depuration takes place in combination with systematic surveillance, shall continue to constitute a considerable primary sector. In this context, the findings of the present study are expected to contribute to defining the safety status of seafood products, in terms of microbial load, in the second largest city in Greece. Additionally, combined application of different detection methods, i.e., both microbiological and molecular ones, seems to be more effective in the detection of public health pathogens. Results revealed from our study can be used by local authorities for the implication of an effective risk assessment for the identification of potential hazards for the public health as well for emphasizing the general safety of the bivalves originating from the Aegean Sea.

## Figures and Tables

**Table 1:** Id, species, market, and date of each sample examined.

ID No	Species	Market	Date	ID No	Species	Market	Date
14	*Callista chione*	Nea Michaniona	3 March 2020	42	*Ostrea edulis*	Kapani	26 June 2020
16	*Callista chione*	Kapani	9 April 2020	56	*Ostrea edulis*	Kapani	7 August 2020
43	*Callista chione*	Nea Michaniona	22 July 2020	68	*Ostrea edulis*	Nea Michaniona	1 September 2020
54	*Callista chione*	Kapani	26 June 2020	70	*Ostrea edulis*	Nea Michaniona	22 July 2020
58	*Callista chione*	Nea Michaniona	10 February 2020	77	*Ostrea edulis*	Nea Michaniona	4 August 2020
59	*Callista chione*	Kapani	7 August 2020	88	*Ostrea edulis*	Kapani	2 November 2020
60	*Callista chione*	Nea Michaniona	1 September 2020	89	*Ostrea edulis*	Nea Michaniona	10 November 2020
64	*Callista chione*	Kapani	4 September 2020	116	*Ostrea edulis*	Kapani	12 January 2021
69	*Callista chione*	Kapani	9 July 2020	200	*Ostrea edulis*	Nea Michaniona	10 December 2020
74	*Callista chione*	Nea Michaniona	15 November 2020	10	*Ruditapes decussatus*	Kapani	28 February 2020
78	*Callista chione*	Kapani	5 October 2020	39	*Ruditapes decussatus*	Kapani	3 June 2020
79	*Callista chione*	Nea Michaniona	4 October 2020	57	*Ruditapes decussatus*	Nea Michaniona	4 August 2020
95	*Callista chione*	Nea Michaniona	10 November 2020	87	*Ruditapes decussatus*	Kapani	5 October 2020
97	*Callista chione*	Kapani	12 November 2020	98	*Ruditapes decussatus*	Nea Michaniona	10 November 2020
104	*Callista chione*	Nea Michaniona	4 August 2020	111	*Ruditapes decussatus*	Kapani	2 December 2020
113	*Callista chione*	Kapani	2 December 2020	112	*Ruditapes decussatus*	Nea Michaniona	10 December 2020
114	*Callista chione*	Nea Michaniona	10 December 2020	1	*Venus verrucosa*	Nea Michaniona	20 January 2020
3	*Mytilus galloprovincialis*	Nea Michaniona	20 January 2020	2	*Venus verrucosa*	Kapani	28 January 2020
4	*Mytilus galloprovincialis*	Kapani	28 January 2020	12	*Venus verrucosa*	Nea Michaniona	3 March 2020
17	*Mytilus galloprovincialis*	Nea Michaniona	16 April 2020	13	*Venus verrucosa*	Kapani	19 March 2020
18	*Mytilus galloprovincialis*	Kapani	21 April 2020	19	*Venus verrucosa*	Kapani	9 April 2020
26	*Mytilus galloprovincialis*	Nea Michaniona	26 May 2020	20	*Venus verrucosa*	Nea Michaniona	10 February 2020
33	*Mytilus galloprovincialis*	Kapani	22 May 2020	24	*Venus verrucosa*	Nea Michaniona	16 April 2020
34	*Mytilus galloprovincialis*	Nea Michaniona	26 May 2020	25	*Venus verrucosa*	Kapani	21 February 2020
45	*Mytilus galloprovincialis*	Kapani	3 June 2020	30	*Venus verrucosa*	Kapani	12 May 2020
46	*Mytilus galloprovincialis*	Nea Michaniona	23 June 2020	35	*Venus verrucosa*	Nea Michaniona	26 May 2020
48	*Mytilus galloprovincialis*	Kapani	26 June 2020	36	*Venus verrucosa*	Kapani	10 June 2020
49	*Mytilus galloprovincialis*	Nea Michaniona	16 June 2020	38	*Venus verrucosa*	Nea Michaniona	30 October 2020
52	*Mytilus galloprovincialis*	Kapani	15 July 2020	40	*Venus verrucosa*	Kapani	3 June 2020
61	*Mytilus galloprovincialis*	Nea Michaniona	1 September 2020	44	*Venus verrucosa*	Nea Michaniona	22 July 2020
62	*Mytilus galloprovincialis*	Kapani	4 September 2020	50	*Venus verrucosa*	Kapani	23 July 2020
71	*Mytilus galloprovincialis*	Nea Michaniona	2 October 2020	51	*Venus verrucosa*	Nea Michaniona	23 June 2020
72	*Mytilus galloprovincialis*	Kapani	2 October 2020	53	*Venus verrucosa*	Kapani	7 August 2020
76	*Mytilus galloprovincialis*	Nea Michaniona	30 October 2020	55	*Venus verrucosa*	Nea Michaniona	4 August 2020
81	*Mytilus galloprovincialis*	Nea Michaniona	22 July 2020	82	*Venus verrucosa*	Kapani	5 October 2020
83	*Mytilus galloprovincialis*	Kapani	7 August 2020	94	*Venus verrucosa*	Kapani	2 November 2020
84	*Mytilus galloprovincialis*	Nea Michaniona	4 August 2020	96	*Venus verrucosa*	Nea Michaniona	10 November 2020
85	*Mytilus galloprovincialis*	Kapani	15 November 2020	99	*Venus verrucosa*	Nea Michaniona	1 September 2020
90	*Mytilus galloprovincialis*	Kapani	16 December 2020	100	*Venus verrucosa*	Kapani	10 September 2020
91	*Mytilus galloprovincialis*	Nea Michaniona	10 December 2020	101	*Venus verrucosa*	Nea Michaniona	25 August 2020
92	*Mytilus galloprovincialis*	Kapani	2 February 2020	103	*Venus verrucosa*	Kapani	12 December 2020
102	*Mytilus galloprovincialis*	Nea Michaniona	10 February 2020	108	*Venus verrucosa*	Kapani	2 December 2020
107	*Mytilus galloprovincialis*	Nea Michaniona	9 March 2021	109	*Venus verrucosa*	Nea Michaniona	10 December 2020
				119	*Venus verrucosa*	Kapani	12 January 2021

**Table 2:** Detected microorganism in each analyzed bivalve. ID code according to Table 1. N.D. corresponds to “not detected”; “−” corresponds to absence of target microorganism; “+” corresponds to presence of target microorganism; *E. coli* is expressed in cfu/100 g.

ID No	*E. coli*	*Salmonella* spp.	*Vibrio* sp.	*Marteilia refringens*	ID No	*E. coli*	*Salmonella* spp.	*Vibrio* sp.	*Marteilia refringens*
1	<18	N.D.	−	−	64	<18	N.D.	+	−
2	<18	N.D.	−	−	68	<18	N.D.	−	−
3	<18	N.D.	−	−	69	<18	N.D.	+	−
4	<18	N.D.	−	−	70	20	N.D.	+	−
10	20	N.D.	−	−	71	<18	N.D.	+	−
12	<18	N.D.	−	−	72	20	N.D.	+	−
13	<18	N.D.	−	−	74	<18	N.D.	+	−
14	20	N.D.	−	−	76	20	N.D.	+	−
16	<18	N.D.	−	−	77	<18	N.D.	+	−
17	20	N.D.	−	−	78	<18	N.D.	+	−
18	<18	N.D.	−	−	79	45	N.D.	+	−
19	<18	N.D.	−	−	81	<18	N.D.	+	−
20	92	N.D.	−	−	82	<18	N.D.	+	−
24	<18	N.D.	−	−	83	<18	N.D.	−	−
25	<18	N.D.	−	−	84	<18	N.D.	−	−
26	<18	N.D.	−	−	85	40	N.D.	−	−
30	45	N.D.	−	−	87	<18	N.D.	+	−
33	20	N.D.	+	−	88	<18	N.D.	+	−
34	<18	N.D.	+	−	89	<18	N.D.	+	+
35	<18	N.D.	+	−	90	<18	N.D.	+	−
36	<18	N.D.	+	−	91	<18	N.D.	−	−
38	<18	N.D.	+	−	92	<18	N.D.	−	−
39	<18	N.D.	+	−	94	<18	N.D.	−	−
40	<18	N.D.	+	−	95	<18	N.D.	+	−
42	<18	N.D.	−	−	96	<18	N.D.	−	−
43	20	N.D.	+	−	97	<18	N.D.	−	−
44	<18	N.D.	+	−	98	<18	N.D.	+	−
45	230	N.D.	+	−	99	<18	N.D.	−	−
46	20	N.D.	+	−	100	<18	N.D.	−	−
48	<18	N.D.	−	−	101	<18	N.D.	+	−
49	20	N.D.	+	−	102	20	N.D.	−	−
50	<18	N.D.	+	−	103	<18	N.D.	−	−
51	20	N.D.	+	−	104	<18	N.D.	−	−
52	<18	N.D.	+	−	107	<18	N.D.	−	−
53	<18	N.D.	+	−	108	130	N.D.	−	−
54	<18	N.D.	+	−	109	45	N.D.	−	−
55	<18	N.D.	+	−	111	<18	N.D.	−	−
56	20	N.D.	+	−	112	20	N.D.	−	−
57	<18	N.D.	+	−	113	<18	N.D.	−	−
58	<18	N.D.	+	−	114	<18	N.D.	−	−
59	<18	N.D.	+	−	116	<18	N.D.	−	+
60	<18	N.D.	+	−	119	<18	N.D.	−	−
61	<18	N.D.	+	−	200	<18	N.D.	−	+
62	2400	N.D.	+	−					

## Data Availability

Data are contained within the article.
